# The use of polarized light in the zonal orientation of the sandhopper *Talitrus saltator* (Montagu)

**DOI:** 10.1186/s40851-023-00207-8

**Published:** 2023-05-18

**Authors:** Alberto Ugolini, Takahiko Hariyama, David C. Wilcockson, Luca Mercatelli

**Affiliations:** 1 Dipartimento Di Biologia, Università Di Firenze, Via Romana 17-19, 50125 Florence, Italy; 2 Institute for NanoSuit Research, Preeminent Medical Photonics Education and Research Center, Hamamatsu University, School of Medicine, 1-20-1 Handayama, Higashi-Ku, Hamamatsu, 431-3192 Japan; 3 Institute of Biological Environmental and Rural Sciences, Aberystwyth University, Penglais, Aberystwyth, SY23 3DA UK; 4 Istituto Nazionale di Ottica – CNR, Largo E. Fermi 6, 50125 Florence, Italy

**Keywords:** *Talitrus saltator*, Skylight polarization, Celestial orientation, Radiance gradient, Spectral gradient

## Abstract

It is well known that the celestial polarization is used as a compass cue by many species of insects and crustaceans. Although it has been shown that the sandhopper *Talitrus saltator* perceives polarized light and possesses an arrangement of the rhabdomeres that could allow e-vector interpretation and utilization, *T. saltator* does not use the e-vector of the skylight polarization as a compass cue when making excursions along the sea–land axis of sandy shores. We performed tests in confined conditions to clarify if skylight polarization is somehow involved in the zonal recovery of *T. saltator*. We observed the directional responses of sandhoppers in a transparent bowl under an artificial sky (an opaline Plexiglas dome). The bowl was covered by a blue gelatin filter with a grey filter (control condition) and a linear polarizing filter (experimental conditions) positioned under the blue one in such a way as to occupy half of the upper surface of the Plexiglas bowl so as to create a linear polarization gradient. Our experiments confirm that *T. saltator* perceives polarized light and highlight that this visual capability determines the perception, or perhaps the increase, of the radiance and/or spectral gradient and their use as compass cues in the zonal orientation. Moreover, our findings confirm that the radiance gradient is used as a chronometric compass orienting reference in the absence of other celestial orienting cues.

**Supplementary Information:**

The online version contains supplementary material available at 10.1186/s40851-023-00207-8.

## Background

Arthropods utilize multiple celestial orienting cues to make efficient excursions, sometimes following a rectilinear path between a particular destination and their homes. During the day, the position of the sun in the sky is generally hierarchically dominant over other celestial orientation cues such as the spectral gradient, radiance gradient, and skylight polarization [1]. However, the relationships in the compass use of these cues may differ between species. For instance, since there is a relationship between the position of the sun and celestial color gradients [2], some arthropods such as bees use spectral gradients of the sky as a compass cue and/or to distinguish between the sun and sky [3], and some ants and butterflies integrate polarization and chromatic cues to obtain compass information [4]. The desert ant *Cataglyphis bicolor* loses its ability to assume a correct homeward direction and perform phototactic responses if the radiance gradient is not perceived together with other sky cues [5]. Some species of dung beetles rely on the gradient of radiance only in combination with the celestial polarization pattern [1]. It is well known that the celestial polarization is used as a compass cue by many species of insects and crustaceans perceived by specialized ommatidia of a particular area of the compound eye (see [6–9] for reviews) and the compass information from the sun and the sky polarization are often integrated at the level of the central complex in the brain (e.g., see [10, 11]).

Several species of supralittoral talitrid amphipods are known for their capacity to use multiple celestial orienting cues to return rapidly to the damp belt of sand or stranded materials in which they spend the hottest hours of the day. This is achieved following the shortest route corresponding to the sea–land axis of the home beach (e.g., see [12, 13]). To do this, the principal daytime celestial compass cue used is the position of the sun, as demonstrated by many classic studies using a mirror to experimentally deflect the solar azimuth [14]; the direction of sandhoppers is shifted by a corresponding angle [12]. Other celestial orientation factors used by talitrids include the spectral and radiance gradients of the sky that can be used as chronometric compass cues [15–18]. However, it should be recognised that differences in the use of celestial orientation factors due to astronomical reasons (e.g., see [19, 20]) are present in different species of supralittoral amphipods living in temperate and equatorial regions.

Although it is well established that skylight polarization is perceived and used as a celestial orientation factor by many crustaceans [6, 8, 9, 21, 22], *T. saltator* does not use of the e-vector to orient to the correct sea–land direction of the home beach [17, 23]. On the other hand, the ability to perceive polarized light has been suggested in some supralittoral amphipods such as *T. saltator* and *Platorchestia platensis* [12, 23, 24], and it is notable also that *T. saltator* possesses an arrangement of the rhabdomeres that might enable e-vector interpretation and utilization [17, 23, 25]. Therefore, we carried out some behavioral tests aimed to clarify the role of the skylight polarization perception in the zonal recovery of *T. saltator*.

## Materials and methods

Adult individuals of *T. saltator* were collected on a sandy beach in the Regional Natural Park of Migliarino, San Rossore, Massaciuccoli, Pisa, Italy (43°40′03″N, 10°20′29″E, sea–land axis of the beach = 265°–85°) in May 2020 and returned to the laboratory. Tests were conducted within 5 days following collection.

In the laboratory, sandhoppers were kept in transparent Plexiglas boxes with wet sand (room temperature 25 ± 2 °C), under a light:dark (L:D) cycle = 12:12 in phase with the natural photoperiod. Food (blotting paper and universal dried food for fish, SERA® Vipan, Heisenberg, Germany) was available ad libitum.

Experiments were conducted in a darkened room, in Florence (43°46′45″N, 11°14′46″E), from 0850 to 0905 h (antimeridian tests), from 1142 to 1215 h (meridian tests) and from 1500 to 1515 h (pomeridian tests) in June 2020. Times represent local solar time.

**Fig. 1:**
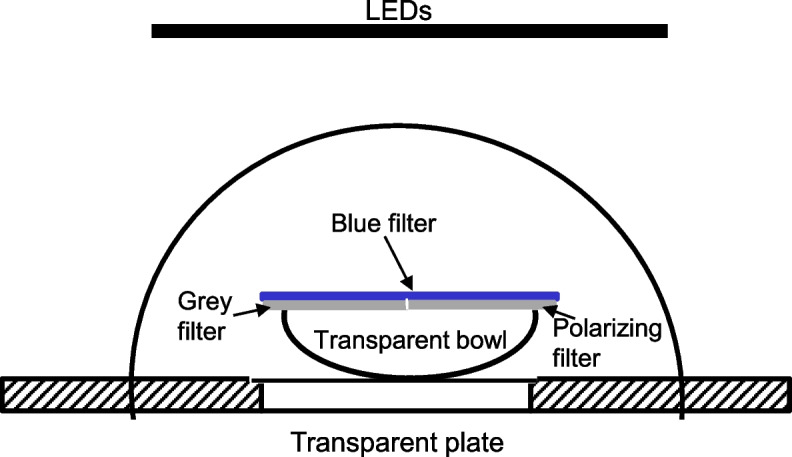
**A**, Schematic representation of the experimental setup

Groups of 8–10 sandhoppers were released in a transparent Plexiglas bowl (Fig. 1, diameter = 18 cm) placed on a horizontal transparent plate (diameter = 28 cm).

The bowl was empty, allowing sandhoppers to jump, walk, or attempt to climb the sides of the bowl. A single direction for each radially oriented individual (with the head pointed toward the outside of the bowl and the longitudinal axis of the body oriented no more than ± 45° from the radius of the bowl) was recorded after 2 min from each release and established from freeze-framed images taken by a camera placed below the bowl. Sandhoppers were released only once. A goniometer was set below the bowl to measure the directions taken by the individuals with respect to 0°, which was set to the north using a compass.

**Fig. 2:**
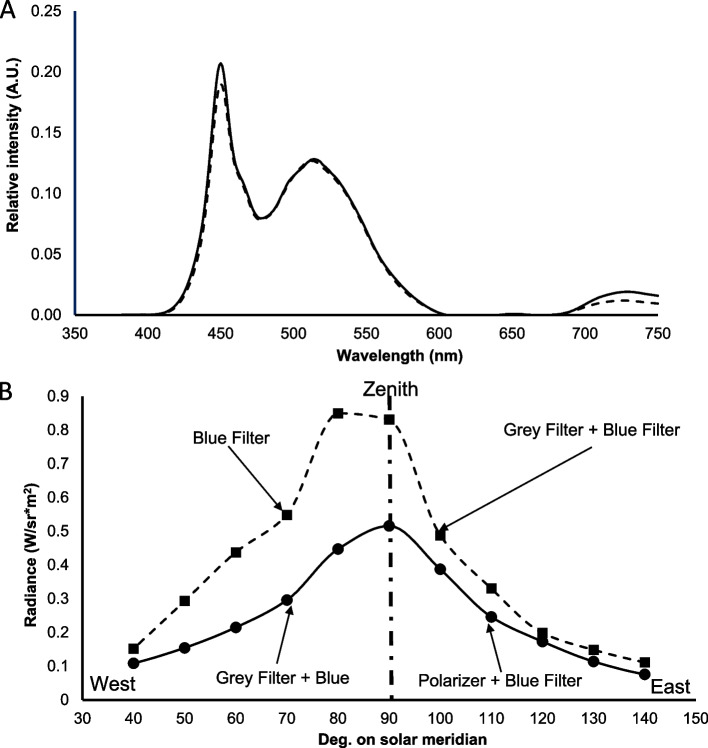
**A**, spectral radiance of the two halves: as grey (neutral density) filter and polarizing filter have similar transmission spectra in the spectral interval allowed by the blue filter, the spectrum of the polarized half (dashed line) is similar to that of the non-polarized one (solid line). **B**, radiance measured along solar meridian of the bowl. Solid line, West and East parts are equalized when a polarized filter and a neutral density grey filter are both present on the East and West area over the bowl, respectively. A radiance gradient is present in the control experiments, when only the grey filter is present on the East part (dashed line). In both cases a blue filter is superimposed

An artificial sky was presented to experimental animals as follows. The bowl was covered with an opaline Plexiglas dome (diameter = 30 cm, Fig. 1) to diffuse the incoming artificial light on the bowl and obscure any potential external visual cues. Since previous experiments showed that some celestial cues used in the orientation of this species are perceived in the UV – blue range [26] and three different peaks of ERG spectral sensitivity curves were observed at 390, 430, and 450 nm [24, 25] we used a blue gelatin filter (no. 118 Light Blue, λmax = 450 nm). A neutral density filter (grey filter no. 209, 0.3 optical density, transmittance = 46%, 400 nm < λ < 600 nm, SpotLight, Milan, Italy), and a linear polarizing filter (HN42, Polaroid – Knight Optical, UK), were each cut in such a way as to occupy half of the upper surface of the Plexiglas bowl. Transmission spectra of all the filters are provided in Fig. S1. The two parts were joined together along the diameter and positioned above the bowl, under the blue filter (Fig. 1). The grey filter was used to equalize the quantity of light perceived by *T. saltator*: in fact, as a polarizing filter transmits just a portion of impinging light (the portion with polarization along the filter polarization axes), a neutral density filter had to be superimposed on the other half of the bowl. A white LED Panel Lamp (100W 12 V, Sumbulbs, spectrum shown in Fig. S1) driven with a controlled power supply (Elind mod. 32DP32) was placed at a distance of 37 cm from the top of the bowl. This arrangement produced irradiation of 150 μW/cm^2^ on the bottom of the bowl, once blue and grey filters were superimposed on one half of the bowl with the blue and polarizing filter on the other half. The spectrum of the LED light, filtered by the blue and the polarizing filter is very similar to the spectrum filtered by the blue and the neutral density filter (Fig. 2A), and thus the spectral information of the two halves was equivalent. The radiance along the solar meridian was measured at 10° intervals using a radiometer (Ophir mod. Nova Display, head PD300UV) with a fiber optic fitted with a lens (objective): the objective was mounted on a miniaturized rotating support with 10° fixed steps; the plane on which the rotation occurred was perpendicular to the horizontal plane. The measurements demonstrate the equality of the two halves (Fig. 2B). Thus, since the spectral information was also very similar in the two halves, the only orientating cue presented was the polarized light on one half of the artificial sky.

**Fig. 3:**
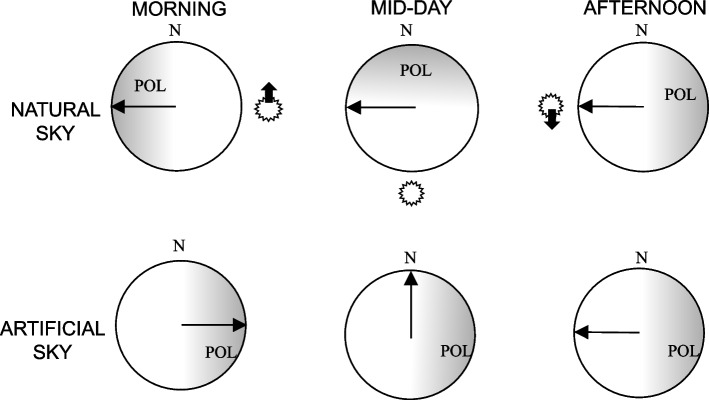
Schematic representation of the maximum intensity of polarization (POL) in natural conditions (the symbol of the sun indicates its azimuth) and under the artificial sky used in these experiments (artificial sun switched off). The black arrows indicate the expected direction of the sandhopper’s orientation

The junction between the grey filter and the polarizer was positioned in a north–south (0°-180°) direction, with the polarizer facing east (90°). This arrangement was kept constant regardless of the time of the experiment during the day in order to randomize the possible influence of any non-uniformity present in the experimental set-up. In this way, the theoretical direction of orientation toward the sea at the home beach (i.e., the expected direction in the case of use of the available orientation factors) changed with the time of day Fig. 3).

Control experiments were carried out using the same apparatus described above except that the grey filter was substituted in place of the polarizer. Therefore, the sandhoppers were allowed to see the blue filter towards the West and the blue filter superimposed on the grey filter towards the East creating a radiance gradient. As depicted in Fig. 2B, the East side of the dashed line was nearly equal to the West side of the solid one (same filter combination); they differed around the zenith, the dashed line being higher because of the acceptance angle of the instrument, into which fell part of the blue light. Figure 2B shows the radiance gradient of controls, as in the West part, with the only blue filter, the sky radiance was much higher than that in the East part. In contrast, when the polarizing filter was equalized by the grey filter, the two sides of the artificial sky exhibited similar radiance (solid line). Control releases were made at the hours corresponding to those of the experimental ones. At the end of the experiments, sandhoppers were returned to the collection site and released.

Circular distributions of animal directions were analyzed using methods proposed in [27]. For each distribution, the length of the mean resultant vector (r) and the mean angle (α) were calculated. To establish whether the distributions differed statistically from uniformity the Rao's spacing test was used (*P* < 0.05 at least). Confidence limits for the mean angle (confidence = 95%) determined whether the mean direction of orientation was in good agreement with the expected direction. Furthermore, we considered the number of individuals exhibiting radial orientation as an indicator of the “difficulty” for sandhoppers in making directional choices. The numbers of radial individuals recorded under the different experimental conditions were compared by using the G-test (*p* < 0.05 at least) [28].

## Results

**Fig. 4:**
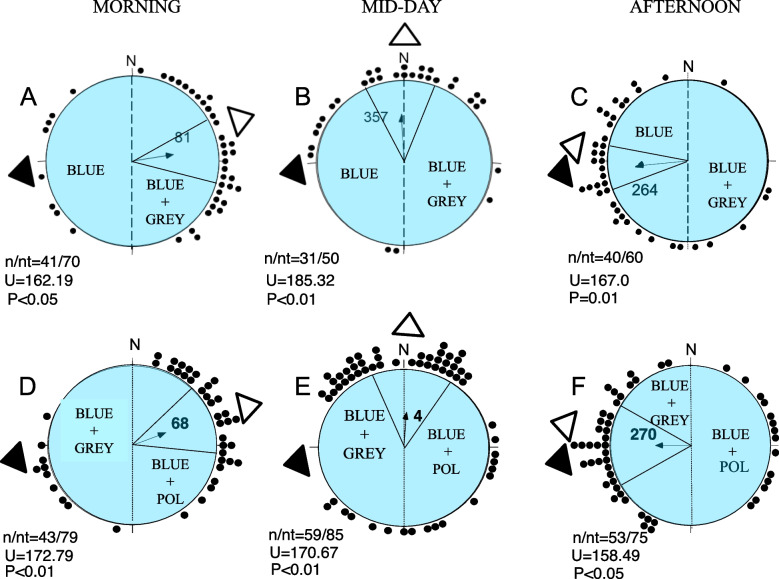
Releases under the dome and artificial sky in the morning (**A**, **D**), mid-day (**B**, **E**), and afternoon (**C**, **F**). **A–C**, blue filter plus grey filter in the eastern half of the artificial sky. **D–F**, **A–C**, blue filter plus grey filter in the western half of the artificial sky and the polarizing filter in the opposite area. The dashed line shows the separation between the grey and the polarizing filter. Each dot represents the direction of one sandhopper; the arrow in each distribution represents the mean vector. The mean angle is also given together with its confidence limits. The black triangles external to the distributions represent the geographic direction of the sea at the home beach. The white triangles indicate the expected direction based on the visual stimuli of the artificial sky. n, number of radially orientated individuals; nt, total number of released sandhoppers. The values of the Rao’s spacing test *U*, with the probability level *P* are also given

In the control conditions, sandhoppers were released under an artificial sky using only the radiance gradient (determined by the presence of the grey filter below half of the blue filter). Under this regime sandhoppers were able to assume the correct orientation towards the expected direction (i.e., the direction of the land–sea axis at the home beach) during the 3 h of the day in which the experiments were carried out (Fig. 4A, B, C). However, the proportion of radially oriented individuals released (112 out of 180 individuals, 62%) suggests some difficulty for the animals to exhibit directionality. Distributions of animals in the absence of the radiance gradient but with the presence of the polarizing filter showed the mean orientations towards the expected direction at all experimented hours of the day (Fig. 4D, E, F) although a tendency towards bimodality was detectable. The number of radially oriented individuals recorded in these experiments (155 out of 239, 65%) was similar to that recorded in the individuals tested under the blue filter with only the grey filter, i.e. under a gradient of radiance (controls: 112 out 180 = 62%,); the difference in radially orienting animals under control and experimental conditions was not statistically significant (G = 0.306, df = 1, *P* = NS, G test).

The comparison between the number of radially oriented individuals registered in previous releases made under natural sky with the sun screened out (data from [17, 25]) and the number of radially oriented sandhoppers registered under the control condition of this study (125 out of 179 = 70%, G = 2.310, df = 1, *P* = NS, G tests) and the experimental one (155 out of 239 = 65%; G = 1.148, df = 1, *P* = NS, G test) does not reach statistical significance.

## Discussion

Our experiments confirm that in *T. saltator* the radiance gradient is used as a chronometric compass orienting reference in the absence of other celestial orienting cues, as previously highlighted (see [15]). This finding contrasts with those for some model insect examples such as the desert ant *Cataglyphis bicolor*, for which the orientation system is disrupted when allowed to orientate using only the radiance gradient [5], and the dung beetle *Scarabaeus lamarki*, which relies on this cue only when combined with the celestial polarization pattern [1] and not for a time-compensated orientation. It has been shown that *T. saltator* does not use the e-vector as a compass cue (see also [23] for *Platorchestia platensis*); however, our results confirm that *T. saltator* is able to perceive polarized light and highlight that this visual capability determines the perception (in our laboratory experiments under artificial light), or perhaps the increase (under natural sky) of the radiance and/or spectral gradient. These, as reported elsewhere, are both used by *T. saltator* as compass chronometric orientation factors [15, 18]. Considering that there is a relationship between celestial color gradients and the position of the sun [2] and that in some species of insects the sun and the skylight cues can be used together [10, 29, 30], it is plausible to hypothesize that in nature this may also be true for *T. saltator*. However, *T. saltator* can exploit the radiance gradient and the spectral gradient as chronometric compass cues, which could both be enhanced by the perception of the skylight polarization, separately and independently from the vision of the solar disk. Since in some insects (e.g., ants and butterflies) there is an integration of polarization and chromatic cues [4] it is possible that the perception of the polarized light of the sky by *T. saltator* might result in a variation of color and/or contrast, thus increasing both the spectral gradient and the radiance gradient. This is despite the separation of color and radiance pathways occurring early in the evolution of the visual system of many species, and despite the traditional definition of color as independent of radiance*.* However, in the aquatic crustacean *Daphnia magna* visual behavior depends on the comparison and integration of multiple wavelength-specific photoreceptor signals, rather than a separation of radiance and wavelength processing [31].

The neuroanatomy of amphipods has not been extensively investigated [32]. Moreover, there is a high disparity in morphology and size of visual neuropils depending on the lifestyle of the different species [33–36]. Unfortunately, the neuroanatomical connections between the different parts of the compound eye and the central nervous system, and in particular to the central complex, are not yet described for *T. saltator.*

In our opinion, the observations reported here are not attributable to the use of the e-vector of the artificial polarization pattern. This assertion is based on previous experiments showing that the interposition and rotation of polarizing filters between the sandhoppers in the bowl and the natural sky cause the animals to remain mostly in the half of the bowl corresponding to the direction of the home beach, but with the percentage of radial individuals practically nil [12, 17, 23].

It is also clear that animals found it more difficult to assume the correct direction of orientation towards the sea under the current experimental conditions than was recorded in previous experiments carried out under natural (223 radial individuals out of 247 = 90%) or artificial (170 radial individuals out of 210 = 81%) sun. We emphasize that our experimental conditions seem to act on the capacity of orientation by *T. saltator* in a similar way based on the vision of celestial cues in the absence of the sun. Since the differences between the number of radially oriented individuals observed under the natural blue sky and controls or experimental conditions are not statistically significant, we hypothesize that 1) the grey filter simulates the natural gradient of radiance, 2) adding the polarizing filter, despite the measured similar radiance between the polarizing and grey filter, created a difference in radiance similar to the natural one under the blue sky and also similar to the radiance gradient shown to the controls. Therefore, the vision of the polarized light in *T. saltator* appears to allow (or increase) the perception of the gradient of radiance and/or color. However, not knowing how the polarized light acts on the visual capacity of *T. saltator*, any further hypotheses would be purely speculative. Therefore, we would like to investigate the relationship between the gradient and polarization of the celestial pattern as the quantity and modality of sensation as the orientation cue used by the sandhopper as a future study.

## Conclusions

Our results confirm that 1) *T. saltator* uses the sky radiance gradient as a chronometric compass orienting reference, and 2) *T. saltator* is able to perceive polarized light, and this highlights that this visual capability determines the perception, or perhaps the increase, of the radiance and/or spectral gradient and their use as compass cues in the zonal orientation of sandhoppers.

## Supplementary Information

**Additional file 1.**

## Data Availability

The datasets used and analyzed during the current study are available from the corresponding author on reasonable request.
