# Supplementary material for: The use of polarized light in the zonal orientation of the sandhopper Talitrus saltator (Montagu)
Source: Zoological Lett. 2023 May 18;9:10. doi: 10.1186/s40851-023-00207-8 (PMC10193715; doi:10.1186/s40851-023-00207-8)
Supplement: Supplementary file 1 — Additional file 1. [file 40851_2023_207_MOESM1_ESM.pptx]

## Slide 1
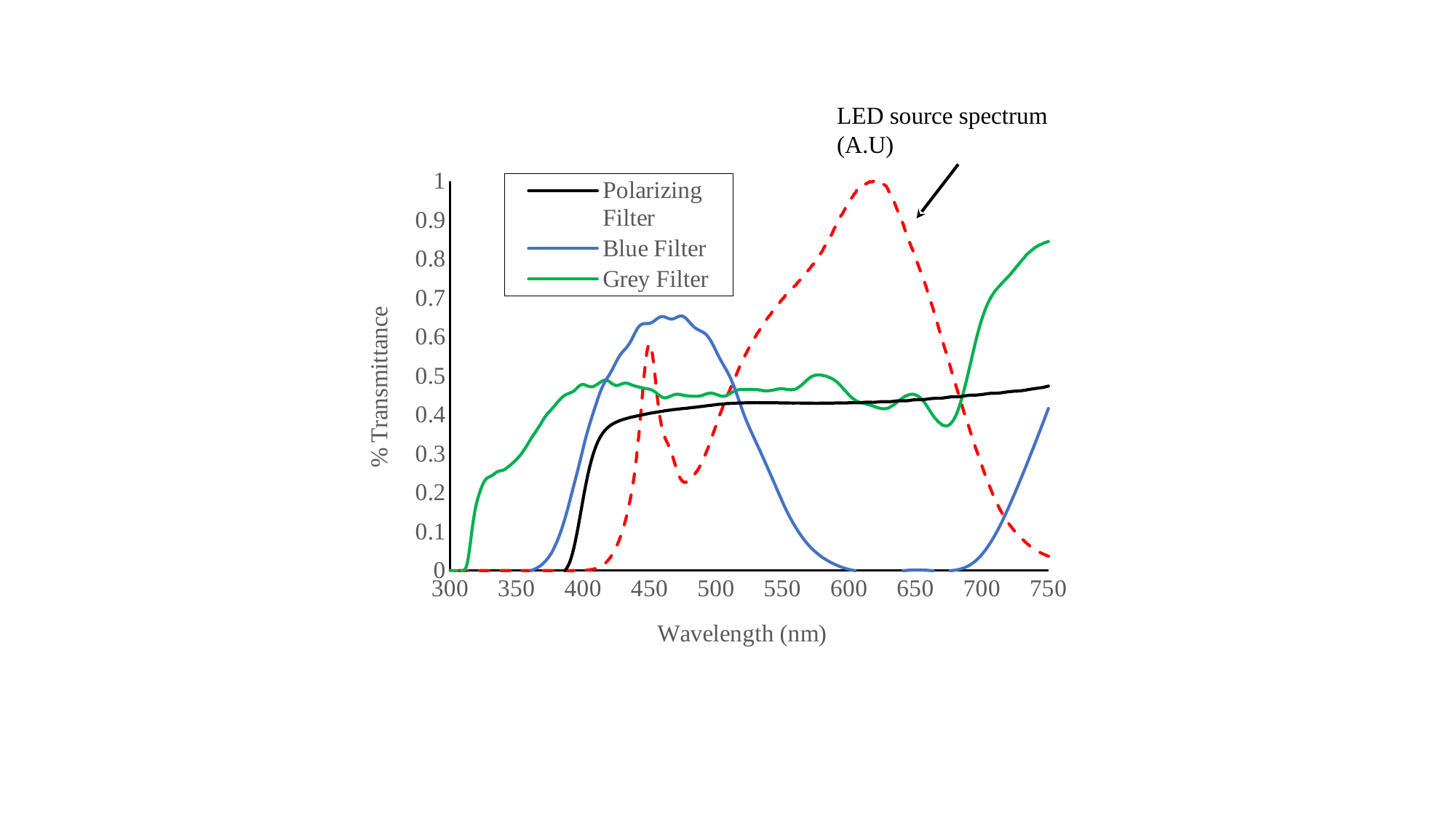

LED source spectrum
(A.U)
### Chart
| Category | Polarizing Filter | Blue Filter | Grey Filter | LED Source |
|---|---|---|---|---|
